# mRNA Levels of Placental Iron and Zinc Transporter Genes Are Upregulated in Gambian Women with Low Iron and Zinc Status

**DOI:** 10.3945/jn.116.244780

**Published:** 2017-05-17

**Authors:** Modou Lamin Jobarteh, Harry J McArdle, Grietje Holtrop, Ebrima A Sise, Andrew M Prentice, Sophie E Moore

**Affiliations:** 1 Medical Research Council Unit The Gambia, Banjul, The Gambia;; 2 Rowett Institute of Nutrition and Health, University of Aberdeen, Aberdeen, United Kingdom;; 3 Biomathematics and Statistics Scotland (BioSS), Aberdeen, United Kingdom; and; 4 Division of Women’s Health, King’s College London, London, United Kingdom

**Keywords:** prenatal, micronutrient, placenta, transporter, mRNA, intervention, iron, zinc, lipid-based nutrient supplement

## Abstract

**Background:** The role of the placenta in regulating micronutrient transport in response to maternal status is poorly understood.

**Objective:** We investigated the effect of prenatal nutritional supplementation on the regulation of placental iron and zinc transport.

**Methods:** In a randomized trial in rural Gambia [ENID (Early Nutrition and Immune Development)], pregnant women were allocated to 1 of 4 nutritional intervention arms: *1*) iron and folic acid (FeFol) tablets (FeFol group); *2*) multiple micronutrient (MMN) tablets (MMN group); *3*) protein energy (PE) as a lipid-based nutrient supplement (LNS; PE group); and *4*) PE and MMN (PE+MMN group) as LNS. All arms included iron (60 mg/d) and folic acid (400 μg/d). The MMN and PE+MMN arms included 30 mg supplemental Zn/d. In a subgroup of ∼300 mother-infant pairs, we measured maternal iron status, mRNA levels of genes encoding for placental iron and zinc transport proteins, and cord blood iron levels.

**Results:** Maternal plasma iron concentration in late pregnancy was 45% and 78% lower in the PE and PE+MMN groups compared to the FeFol and MMN groups, respectively (*P* < 0.001). The mRNA levels of the placental iron uptake protein transferrin receptor 1 were 30–49% higher in the PE and PE+MMN arms than in the FeFol arm (*P* < 0.031), and also higher in the PE+MMN arm (29%; *P* = 0.042) than in the MMN arm. Ferritin in infant cord blood was 18–22% lower in the LNS groups (*P* < 0.024). Zinc supplementation in the MMN arm was associated with higher maternal plasma zinc concentrations (10% increase; *P* < 0.001) than in other intervention arms. mRNA levels for intracellular zinc-uptake proteins, in this case zrt, irt-like protein (ZIP) 4 and ZIP8, were 96–205% lower in the PE+MMN arm than in the intervention arms without added zinc (*P* < 0.025). Furthermore, mRNA expression of ZIP1 was 85% lower in the PE+MMN group than in the PE group (*P* = 0.003).

**Conclusion:** In conditions of low maternal iron and in the absence of supplemental zinc, the placenta upregulates the gene expression of iron and zinc uptake proteins, presumably in order to meet fetal demands in the face of low maternal supply. The ENID trial was registered at www.controlled-trials.com as ISRCTN49285450.

## Introduction

Adequate maternal nutrition before and during pregnancy is important for optimal pregnancy outcomes. However, in sub-Saharan Africa, many women of reproductive age, especially those living in poor rural settlements, are recognized to have low dietary intakes of essential nutrients, contributing to deficiencies (1). These nutritional deficiencies are exacerbated during pregnancy, causing risk to both the mother and her unborn infant. Iron and zinc deficiencies are very common. Approximately 56% of women in sub-Saharan Africa are estimated to be anemic, mainly as a result of iron deficiency (2). Although the true extent of zinc deficiency is unknown, ≤80% of women might be at risk of zinc deficiency because of habitual low dietary zinc intake (3). Maternal nutritional deficiency is associated with poor pregnancy outcomes, including low birth weight, small for gestational age, and prematurity (4, 5).

Prevention of nutritional deficiency during pregnancy is thus an important public health challenge in such settings. The most widely used strategy is the daily supplementation of iron (60 mg) and folic acid (400 μg) (FeFol). Because many women in resource-poor settings suffer multiple micronutrient (MMN) deficiencies, UNICEF/WHO/United Nations University (UNU) developed an MMN supplement for pregnant and lactating mothers (6). However, the evidence supporting the advantages of an MMN supplement over FeFol remains limited (7, 8) and alternate ways to improve the nutritional status of pregnant and lactating women are being explored, such as with the use of supplementary lipid-based nutrient supplements (LNSs) (9–11). LNSs are a recently developed group of products shown to be a very effective option for the community-based treatment of severe malnutrition if a high dose is given (e.g., reference 12). They may also provide a route for the delivery of micronutrients that may be preferable to and more efficacious than other products (13). In trials of nutritional supplementation, LNSs can also be designed to include varying amounts of protein and energy (from small to large quantities).

Despite the increasing focus on different intervention strategies during pregnancy, the impact of maternal nutritional status on the regulation of nutrient transport in the human placenta, and hence on fetal supply, is largely unknown. During pregnancy a fetus is entirely dependent on placental transport for its nutrition; nutrients are transported via specialized nutrient transporters located on the placental microvillous and basal membranes (14). Evidence from animal studies indicates that, in the presence of low maternal dietary supply of micronutrients, signals from the fetus can upregulate the expression and activity of micronutrient transporters in the placenta to try to meet its nutritional needs (15, 16). Conversely, the expression and activity of these transporters are lower in the placenta when maternal dietary supply is adequate. Thus placental nutrient transporter response to maternal nutritional status is key in assessing fetal nutritional accretion during pregnancy.

In a marginally nourished rural population, we investigated the effect of nutritional supplementation during pregnancy on maternal iron and zinc status, the expression of mRNA of iron and zinc transporter proteins in the placentas, and the impact on fetal cord blood iron status.

## Methods

### Study population

This study was nested within the prenatal arm of a randomized trial investigating the effect of prenatal and infancy nutritional supplementation on infant immune development in rural Gambia [the ENID (Early Nutrition and Immune Development) trial; ISRCTN49285450] (17). The ENID trial was conducted in the West Kiang region of The Gambia, a rural subsistence community that farms primarily groundnuts and rice. West Kiang is ∼145 km (∼90 miles) inland from Banjul, the capital of The Gambia, and has a stable resident population of ∼14,000 inhabitants distributed among 36 villages. The Gambia has a tropical climate with 2 main seasons: a hot, rainy season (June to October) and a cooler, dry season (November to May). The beginning of the dry season is characterized by food availability, whereas the wet season is characterized by food scarcity, increased energy expenditure from farming activities, and an increased incidence of infectious diseases (18).

### Prenatal intervention and assessments

The ENID trial was a randomized, partially blind trial of nutritional supplementation in pregnant women and their infants in the West Kiang region of the Gambia. This nested substudy focuses on only the pregnancy intervention, and so we do not describe the infants’ intervention. Full details of the ENID trial protocol are published elsewhere (17). Briefly, women between 18 and 45 y of age were invited to participate through informed consent. Each month after enrollment, participating women were visited by a member of the study team and completed a short questionnaire asking about the date of their last menstrual period. Women confirmed as being <20 wk pregnant and with a singleton pregnancy were randomly assigned into the trial.

Upon entering the trial, eligible pregnant women were randomly assigned to 1 of 4 intervention arms: *1*) FeFol supplementation provided 60 mg Fe/d and 400 μg folic acid/d. This arm represents the standard of care during pregnancy, per Gambian government guidelines. *2*) MMNs were provided as a combination of 15 micronutrients specifically formulated for use during pregnancy by UNICEF/WHO/UNU. However, unlike the UNICEF/WHO/UNU formulation, a single MMN tablet in the ENID trial provided twice the RDA of all nutrients except for iron and folate, which were kept at 60 mg/d and 400 μg/d, respectively, as in the FeFol arm. The decision to supplement at twice the RDA was based on evidence from West Africa suggesting that MMNs supplemented at twice the RDA are more effective with regard to birth outcomes (19). Both the FeFol and MMN supplements were formulated as tablets and manufactured by Scanpharm. *3*) Protein-energy (PE), an LNS, provided the same amounts of iron and folic acid as in the FeFol arm, but with the addition of energy, protein, and lipids. *4*) PE and MMNs (PE+MMN), a micronutrient-fortified LNS supplement providing the same amounts of micronutrients as in the MMN arm (including FeFol), in addition to the energy, protein, and lipid content. The 2 LNS products were manufactured by Valid Nutrition, Nairobi, Kenya. The composition of each of the 4 prenatal nutritional intervention arms is detailed in **Table 1**. Details of the procedure randomizing and allocating women to the intervention arms were outlined in the published trial protocol (17).

**TABLE 1:** Nutritional composition of daily intake of pregnancy supplements in the Early Nutrition and Immune Development trial^1^

	Tablets		LNS	
	FeFol	MMN	PE	PE+MMN
Iron, mg	60	60	60	60
Folic acid, μg	400	400	400	400
Vitamin A, μg	—	1600	—	1600
Vitamin D, IU	—	400	—	400
Vitamin E, mg	—	20	—	20
Vitamin C, mg	—	140	—	140
Thiamin, mg	—	2.8	—	2.8
Riboflavin, mg	—	2.8	—	2.8
Niacin, mg	—	36	—	36
Vitamin B-6, mg	—	2.8	—	2.8
Vitamin B-12, μg	—	5.2	—	5.2
Zinc, mg	—	30	—	30
Copper, mg	—	4	—	4
Selenium, μg	—	130	—	130
Iodine, μg	—	300	—	300
Energy, kcal	—	—	746	746
Protein, g	—	—	20.8	20.8
Lipids, g	—	—	52.6	52.6

1 FeFol, iron and folate; LNS, lipid-based nutrient supplement; MMN, multiple micronutrient; PE, protein energy.

### Data and blood sample collection during pregnancy

At enrollment (“booking”) and at 20 and 30 wk of gestation, women attended the Medical Research Council (MRC) Keneba clinic for a detailed antenatal assessment. A standard antenatal check, including blood pressure measurement and hemoglobin and urine analyses, was performed at each visit. In addition, data on maternal anthropometry, including weight, height, waist and hip circumferences, and midupper arm circumference, were collected. Gestational age was determined by ultrasound. All measurements were made using standard, regularly validated equipment and following the relevant standard operating procedures. At each visit, after an overnight fast, a sample of venous blood (20 mL) was collected in trace element–free tubes (Sarstedt AG & Co), and aliquots of plasma were prepared and stored for subsequent analysis.

### Sample collection at birth

In this community, the majority of women choose to deliver in their homes with the support of a traditional birth attendant. Through a network of field workers, deliveries in both homes and health centers were attended, when possible, by a midwife or traditional birth attendant. For home deliveries, field assistants residing in the village attended the delivery to collect cord blood and the placenta. The sample of cord blood (≤15 mL) was collected immediately after delivery. The placenta was then sealed in a sterile plastic bag and transported on ice, with the cord blood, to the MRC Keneba laboratory for processing (20).

Within 72 h of delivery, a trained midwife conducted neonatal anthropometry, including weight, length, midupper arm circumference, and head circumference.

### Laboratory analysis

#### Assessment of maternal iron and zinc status during pregnancy.

Whole-blood samples collected from the women at booking and 20 and 30 wk of gestation were used to assess hemoglobin concentration using a Medonic hematology analyzer (Clinical Diagnostic Solutions, Inc.). Plasma samples from the same time points were later assessed for the following panel of iron indicators: ferritin, serum soluble transferrin receptor (sTfR), hepcidin, transferrin, unbound iron-binding capacity (UIBC), and iron. Plasma ferritin, transferrin, iron, and UIBC were quantified using an automated multianalyte analyzer (COBAS INTEGRA 400 plus; Roche Diagnostics). Plasma hepcidin was quantified by competitive ELISA [Hepcidin-25 (human) ELISA kit; Bachem), according to the manufacturer’s instruction. Plasma sTfR was quantified using a Quantikine IVD ELISA kit (R&D Systems), according to the manufacturer’s instructions. Cord blood samples were analyzed for only hemoglobin, iron, and ferritin.

Only maternal blood samples collected at 30 wk of gestation were analyzed for plasma zinc, using an inductively coupled plasma mass spectrometer (7700 series; Agilent Technologies). Before inductively coupled plasma mass spectrometry, the plasma samples were predigested with nitric acid. This involved adding 40 μL ultragrade nitric acid (60% w/w; Merck) and 10 μL hydrogen peroxide (30% w/w; Sigma-Aldrich) to 50 μL plasma. The mixture was heated at 80°C for 40 min on a hot block, allowed to cool to room temperature, then diluted with ultrapure water (1:20). Trace element control (trace elements serum 1; Seronorm; Sero AS) was analyzed alongside the samples as a quality control. Five replicated measures of the samples, control, and standards were analyzed and the mean taken as the concentration of zinc in parts per billion. Measures in parts per billion were converted to micromoles per liter, with the molecular weight of zinc taken as 65.38 g/mol.

#### Placental sample processing.

Placental samples were processed according to a standard protocol adapted from Pasupathy et al. (21). Upon arrival at the laboratory, the placentas were weighed using a digital scale, and the length, breadth, and thickness were measured with a graduated ruler. Four small sections (∼1–2 mm) of the placental tissues were dissected from the maternal side and washed in sterile cold (4°C) PBS to remove excess blood. From these, another 4 small sections (∼50 mg) were cut and fixed in RNALater solution (Qiagen); these samples were stored at −70°C and sent frozen to the Rowett Institute of Nutrition and Health, Aberdeen, United Kingdom, for RNA extraction and RT-PCR.

### RNA isolation and RT-PCR

Total RNA was isolated from the placenta by homogenizing 50–100 mg tissue in cold TRI Reagent (Helena Biosciences) on wet ice using a Dounce homogenizer. RNA was precipitated overnight in isopropanol and the precipitated RNA was dissolved in diethyl pyrocarbonate–treated water. RNA quantity and purity were measured spectrophotometrically by measuring OD at absorbance ratios of 260/280 and OD 260/230, respectively, using a NanoDrop 2000c spectrophotometer (Thermo Fisher Scientific). RNA integrity was determined using an Agilent 2100 Bioanalyzer (Agilent Technologies), according to the manufacturer’s instructions. Samples with RNA integrity number ≤4 were not included in the final analysis because degraded RNA might affect the quality of the data (20).

RNA (1 μg) was treated with DNase I, Amp Grade (Invitrogen), according to the manufacturer’s instructions. Total RNA (200 ng) was reverse-transcribed into cDNA using a TaqMan MicroRNA Reverse Transcription Kit (Applied Biosystems), according to the manufacturer’s instructions. The reverse-transcription reaction was carried out using a PTC-100 thermocycler (MJ Research Inc.), under the following conditions; 25°C for 10 min, 37°C for 60 min, 95°C for 5 min, and hold at 4°C.

RT-PCR reactions were carried out using a 7500 Fast RT-PCR system (Applied Biosystems), and each reaction contained 1.5 μL of primer (Qiagen), 7.5 μL Power SYBR Green PCR Mastermix (Applied Biosystems), and 5 μL cDNA in a 15-μL reaction. The PCR reactions were set at 95°C for 15 min, 60°C for 10 min (40 repeats), an added dissociation stage (95°C for 15 min), then 60°C for 1 min, and finally 95°C for 15 min (1 repeat). The results were obtained as cycle threshold and a single melt curves were obtained for all samples, indicating that a single PCR product was generated. The cycle threshold values were obtained for both the gene of interest (**Supplemental Table 1**) and the normalizing gene, ubiquitin (QT00234430; Qiagen). Gene expression was normalized to ubiquitin, and the relative expression of the gene of interest was expressed as 2^−ΔCT^.

### Statistical analysis

The primary outcomes of this substudy were maternal iron and zinc status during pregnancy and mRNA expression of placental iron and zinc transporter proteins. The outcomes according to nutritional intervention arm were analyzed on an intent-to-treat basis, that is, regardless of adherence to treatment. This study was powered to detect a treatment effect of ≥15% on maternal iron and zinc status, assuming a between-subject spread of 25%, at a power of 90% and a significance level of 5%. This required 75 mothers/treatment arm.

Data regarding maternal iron status were obtained at booking and weeks 20 and 30 of gestation, and were initially analyzed as a repeated-measures 2-factor ANOVA. Because the interaction between nutritional supplementation and week of gestation was significant (*P* < 0.001) for all outcomes, subsequent analyses were based on 1-factor ANOVA for one time point at a time. Maternal characteristics at enrollment and placental and neonatal data were analyzed by 1-factor ANOVA.

All ANOVAs were adjusted for the following competing effect modifiers or confounders (i.e., variables that neither affected the intervention nor were affected by the intervention, but that could influence the outcomes of interest): gestational age and maternal BMI at booking, season of birth, and maternal parity. For both the repeated-measures 2-factor ANOVA and the 1-factor ANOVA per time point, the maternal iron markers were also adjusted for C-reactive protein, which was log-transformed to avoid the adjustments being driven by outliers. The mean values presented have been adjusted for these confounders. When the effect of nutritional supplementation was significant (*P* < 0.05), post hoc *t* tests, based on the ANOVA findings, were used to explore differences between the nutritional treatment arms.

The assumptions of constant variation and normality required for ANOVA were assessed from visual inspection of residual plots; where necessary, data were log-transformed before proceeding with the analysis described above. These data are presented as back-transformed means (adjusted for confounders) and their corresponding back-transformed 95% CIs. Binary (yes or no) data such as preterm birth were analyzed using contingency tables; when significant this analysis was followed by post hoc comparisons based on normal approximation. All statistical analyses were carried out using Genstat 17.1 statistical software (VSN International Ltd.); however, GraphPad Prism software was used to present the graphs.

### Ethics and governance

The ENID trial and the placental substudy were approved by a joint Gambia Government/MRC unit, The Gambia Ethics Committee (project no. SCC1126v2 and L2010.93, respectively). Written informed consent was obtained from all women before enrollment into the trial. The trial observed Good Clinical Practice standards and the current version of the Declaration of Helsinki.

## Results

### 

#### Recruitment and attrition.

**FIGURE 1:**
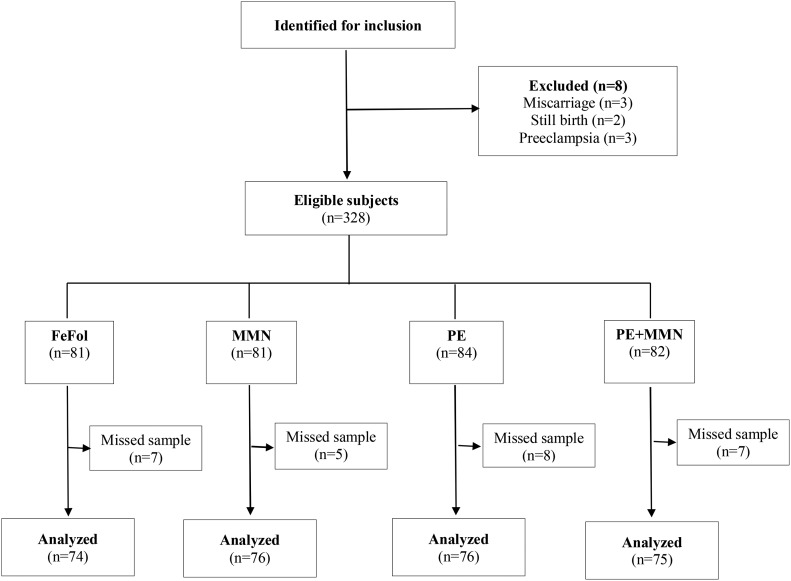
Subject flowchart summarized according to Consolidated Standards of Reporting Trials. FeFol, iron and folic acid; MMN, multiple micronutrient; PE, protein energy.

Between April 2010 and June 2013 a total of 875 women were enrolled in the ENID trial. For this substudy, 336 women who delivered within a 14-mo period (October 2010 to December 2011) were identified for possible inclusion in this substudy. This included all women who delivered while in the ENID trial during the 14-mo period of this substudy. Of the 336 women targeted for inclusion, 8 women were excluded: 3 participants miscarried, 2 participants had a stillbirth, and 3 had preeclampsia. A total of 27 women were not included in the placental substudy (FeFol group: *n* = 7; MMN group: *n* = 5; PE group: *n* = 8; and PE+MMN group: *n* = 7) because of missing placental samples. Placental samples were missed mainly when a mother was referred for hospital delivery outside the West Kiang study area. The final analysis included the 301 mother-infant pairs with placental samples collected (**Figure 1**). No statistically significant differences were found in the maternal characteristics at booking (age, BMI, and gestational age) between the women included in this substudy and those not included (data not shown).

#### General subject characteristics at booking.

The mean ± SD parity of participants was 4.18 ± 2.57 (range: 0–11); mean ± SD age was 30.2 ± 6.22 y (range: 18–45 y); and mean ± SD BMI (in kg/m^2^) was 20.9 ± 3.22 (range: 13.9–36.6) at booking. In this group of women, 20.3% were underweight (BMI <18.5), 70.4% had a normal BMI (19–24), 6.6% were overweight (BMI 25–30), and 2.7% were obese (BMI >30). The mean ± SD gestational age at booking was 14.0 ± 3.24 wk. The prevalence of anemia (hemoglobin <11.0 g/dL) was 31%; iron deficiency (ferritin <12 μg/L), 28.6%; and iron deficiency anemia (hemoglobin <11.0 g/dL and ferritin <12 μg/L), 12.3%.

#### Maternal and newborn characteristics by supplementation arm.

Within the subgroup of women included in this study, maternal anthropometric measures and gestational age at booking did not differ between the intervention arms (**Table 2**). Further, no difference was found in the prevalence of low birth weight or preterm birth between the intervention arms (Table 2). In addition, neonatal anthropometry and placental measures did not differ between the intervention arms (Table 2).

**TABLE 2:** Maternal characteristics at enrollment and at 20 and 30 wk of gestation, and neonatal characteristics at birth^1^

Characteristic	FeFol group(*n* = 75)	MMN group(*n* = 75)	PE group(*n* = 76)	PE+MMN group(*n* = 75)	*P* value^2^
Maternal					
Age, y	30.6 ± 6.4	29.6 ± 5.5	30.6 ± 6.4	30.2 ± 6.7	0.74
Weight, kg	55.1 ± 7.9	54.5 ± 10.0	54.9 ± 9.1	55.8 ± 9.9	0.83
Height, cm	162.3 ± 6.5	161.8 ± 5.8	162.6 ± 6.0	161.7 ± 6.0	0.75
BMI, kg/m^2^	20.9 ± 2.7	20.8 ± 3.5	20.7 ± 3.2	21.3 ± 3.5	0.65
MUAC, cm	26.8 ± 2.7	26.4 ± 3.5	26.6 ± 3.1	26.8 ± 3.0	0.82
Gestational age, wk	14.2 ± 3.2	14.2 ± 3.5	13.9 ± 3.3	13.8 ± 2.9	0.78
Gestational weight gain, kg	4.91 ± 2.90	5.12 ± 3.08	5.29 ± 2.80	5.35 ± 2.95	0.80
Intervention period, wk	26.3 ± 3.2	26.1 ± 3.6	26.3 ± 3.8	26.4 ± 3.4	0.96
Prevalence of anemia,^3^ *n* (%)					
At booking	21 (28.0)	22 (29.3)	25 (32.9)	25 (33.3)	0.87^4^
At 20 wk of gestation	20 (26.7)	25 (33.3)	28 (36.8)	25 (33.3)	0.60^4^
At 30 wk of gestation	32 (42.7)^a^	26 (34.7)^a^	53 (69.7)^b^	46 (61.3)^b^	<0.001^4^
Placenta					
Weight, g	515 ± 100	523 ± 91	525 ± 97	513 ± 103	0.84
Length, cm	18.6 ± 1.9	18.5 ± 1.3	18.7 ± 2.0	18.9 ± 2.1	0.73
Breadth, cm	16.6 ± 1.6	16.7 ± 1.4	16.5 ± 1.9	16.5 ± 1.5	0.94
Thickness, cm	2.12 ± 0.43	2.20 ± 0.48	2.15 ± 0.39	2.17 ± 0.56	0.76
Placental weight–to–birth weight ratio	0.167 ± 0.04	0.173 ± 0.03	0.178 ± 0.03	0.167 ± 0.03	0.069
Neonatal					
Weight, g	3058 ± 402	3040 ± 396	2979 ± 383	3021 ± 420	0.64
Length, cm	49.7 ± 1.5	49.5 ± 1.7	49.4 ± 2.0	49.7 ± 2.0	0.63
Head circumference, cm	33.6 ± 1.4	33.5 ± 2.0	33.2 ± 1.3	33.5 ± 1.7	0.47
Arm circumference, cm	10.9 ± 0.8	10.6 ± 0.9	10.6 ± 0.7	10.7 ± 0.9	0.29
Gestational age, wk	40.5 ± 1.3	40.5 ± 1.5	40.2 ± 1.9	40.2 ± 1.5	0.48
Low birth weight,^5^ *n* (%)	5 (6.7)	7 (9.3)	7 (9.2)	9 (12.0)	0.74^4^
Preterm birth,^5^ *n* (%)	0 (0.0)	1 (1.3)	1 (1.3)	2 (2.7)	0.57^4^
Ponderal Index, g/cm^3^ × 100	2.50 ± 0.36	2.51 ± 0.29	2.48 ± 0.36	2.46 ± 0.29	0.72

1 Values are means ± SDs unless otherwise indicated. Labeled values in a row without a common superscript letter differ (*P* < 0.05). FeFol, iron and folate; MMN, multiple micronutrient; MUAC, midupper arm circumference; PE, protein energy.

2 Obtained from 1-factor ANOVA. Placenta and neonatal outcomes were adjusted for gestational age and maternal BMI at booking, season of birth, and maternal parity; their means have been adjusted accordingly (no adjustments were made for maternal outcomes).

3 Anemia cutoff was hemoglobin <11.0 g/dL at booking and 30 wk of gestation, and hemoglobin <10.5 g/dL at 20 wk of gestation.

4 Analyzed with contingency tables.

5 The cutoff for low birth weight was <2500 g; the cutoff for preterm birth was <37 wk of gestation.

#### Effect of supplementation on maternal iron and zinc status during pregnancy.

**FIGURE 2:**
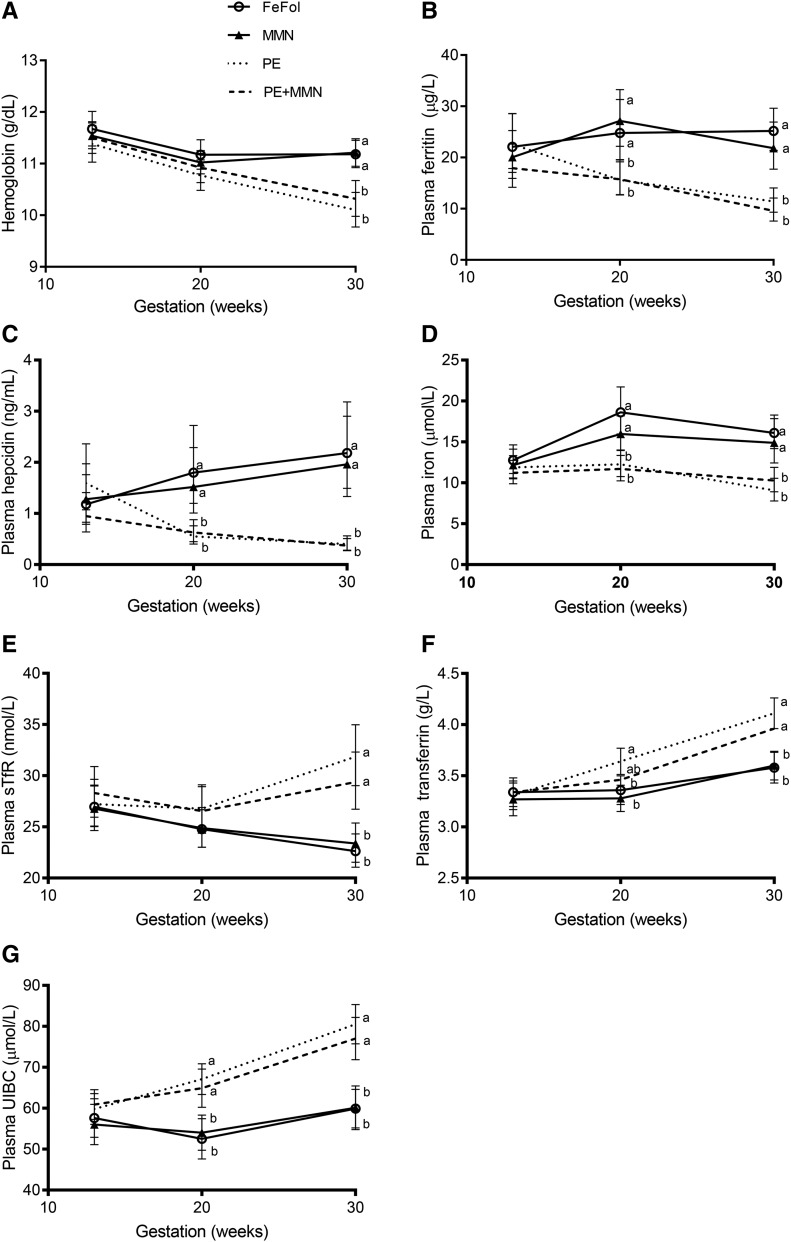
Maternal plasma concentrations of hemoglobin (A), ferritin (B), hepcidin (C), iron (D), sTfR (E), transferrin (F), and UIBC (G) at booking and 20 and 30 wk of pregnancy, by intervention arm. Data were analyzed by repeated-measures 2-factor ANOVA, adjusted for gestational age and maternal BMI at booking, season of birth, maternal parity, and log-transformed C-reactive protein. Significance of supplementation (*P_s_*), week (*P_w_*), and its interaction (*P_sw_*) for each concentration are as follows: hemoglobin: *P_s_* = 0.023, *P_w_* < 0.001, *P_sw_* < 0.001; ferritin: *P_s_* < 0.001, *P_w_* < 0.001, *P_sw_* < 0.001; hepcidin: *P_s_* < 0.001, *P_w_* < 0.001, *P_sw_* < 0.001; iron *P_s_* < 0.001, *P_w_* < 0.001, *P_sw_* < 0.001; sTfR: *P_s_* = 0.019, *P_w_* = 0.005, *P_sw_* < 0.001; transferrin: *P_s_* = 0.003, *P_w_* < 0.001, *P_sw_* < 0.001; and UIBC: *P_s_* < 0.001, *P_w_* < 0.001, *P_sw_* < 0.001. Then, for each time point, data were analyzed by 1-factor ANOVA adjusted for the variables listed above. When the effect of supplementation was significant, this ANOVA was followed by the post hoc *t* test. Transferrin and UIBC are presented as adjusted means ± SEMs (*n* = 71–75). Hemoglobin, ferritin, hepcidin, and iron were log-transformed before analysis and are presented as the back-transformed adjusted means and their corresponding back-transformed 95% CIs (*n* = 66–75). Means at a given time point without a common letter differ, *P* < 0.05. FeFol, iron and folic acid; MMN, multiple micronutrient; PE, protein energy; sTfR, serum soluble transferrin receptor; UIBC, unbound iron-binding capacity.

No difference was observed in any of the measured indicators of maternal iron status (hemoglobin, plasma ferritin, iron, hepcidin, sTfR, transferrin, or UIBC) at booking. By 20 wk of gestation, no difference was detected for mean hemoglobin or sTfR concentrations. However, significant effects of intervention (*P* < 0.001) were observed with all other markers, according to supplement type [i.e., LNSs (PE and PE+MMN groups) compared with tablets (FeFol and MMN groups)]: plasma ferritin, iron, and hepcidin were significantly lower in the LNS arms (PE and PE+MMN) compared with the FeFol and MMN arms (*P* < 0.003; **Figure 2**), whereas plasma transferrin and UIBC were significantly higher in the LNS arms (*P* < 0.003; Figure 2) compared with the FeFol and MMN arms. At week 30 of pregnancy, all maternal iron status indicators showed significant effects of intervention (*P* < 0.001); mean hemoglobin, plasma ferritin, iron, and hepcidin concentrations were all significantly lower in the LNS arms than in the FeFol and MMN arms (*P* < 0.001; Figure 2), whereas mean plasma sTfR, transferrin, and UIBC values were all significantly higher in the LNS arms than in the FeFol and MNN arms (*P* < 0.001; Figure 2).

The prevalence of maternal anemia at booking and at 20 wk of gestation was not statistically significant between the intervention arms (Table 2). By 30 wk of gestation, however, maternal anemia was more prevalent in the LNS intervention arms than in the FeFol and MMN arms (*P* < 0.001; Table 2).

**FIGURE 3:**
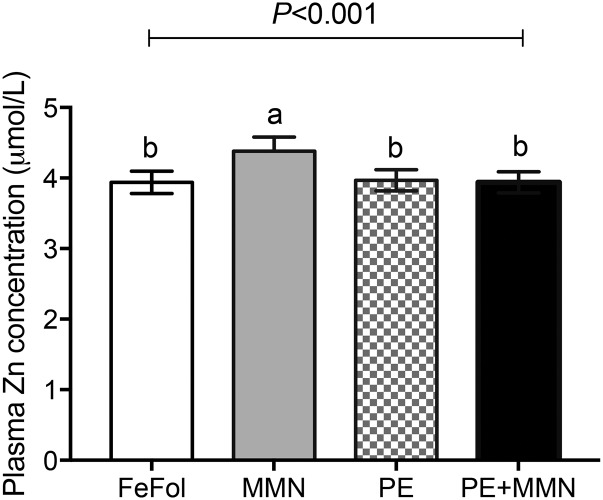
Maternal plasma zinc concentrations at 30 wk of gestation, by intervention arm. Data were analyzed by 1-factor ANOVA, adjusted for gestational age and maternal BMI at booking, season of birth, and maternal parity. Values are adjusted means ± SEMs (*n* = 71–74). Means without a common letter differ, *P* < 0.05 (post hoc *t* test). FeFol, iron and folic acid; MMN, multiple micronutrient; PE, protein energy.

At week 30 of gestation, mean plasma zinc concentrations were higher in the MMN intervention arm than in the FeFol, PE, and PE+MMN arms (*P* < 0.001 for all comparisons; **Figure 3**).

#### Effect of supplementation on mRNA expression of genes encoding for placental iron transporter proteins.

**FIGURE 4:**
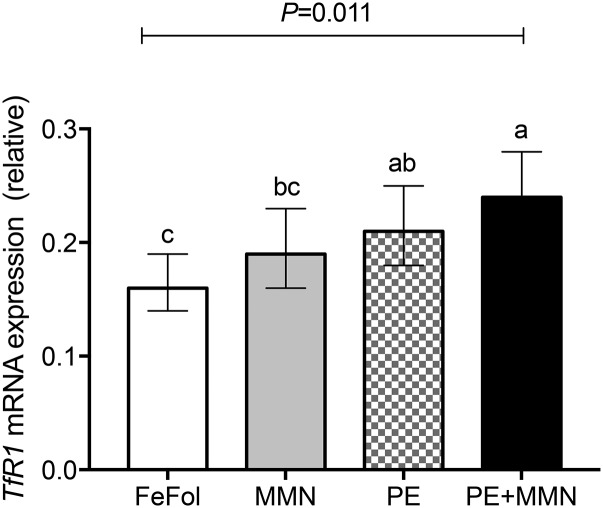
Placental mRNA expression of *TfR1* relative to the ubiquitin housekeeper, by intervention arm. Placental samples with an RNA integrity number >4 were analyzed. Log-transformed data were analyzed by 1-factor ANOVA, adjusted for gestational age and maternal BMI at booking, season of birth, and maternal parity. Values are back-transformed adjusted means and their corresponding back-transformed 95% CIs (*n* = 69–73). Means without a common letter differ, *P* < 0.05. FeFol, iron and folic acid; MMN, multiple micronutrient; PE, protein energy; *TfR1*, transferrin receptor 1.

The mRNA expression of genes responsible for the transport of iron across the placenta, including *TfR1*, divalent metal ion transporter 1 (*DMT1*), ferroportin 1 (*FPN1*), hepcidin, and zyklopen (*ZP*), were measured. Transferrin receptor 1 (*TfR1*) mRNA expression was 30% (95% CI: 3%, 66%; *P* = 0.031) and 49% (95% CI: 17%, 90%; *P* = 0.001) higher in the LNS arms (PE and PE+MMN) than in the FeFol arm (**Figure 4**). Furthermore, *TfR1* mRNA expression was 29% (95% CI: 1%, 64%; *P* = 0.042) higher in the PE+MMN arm than in the MMN arm. However, placental *TfR1* mRNA expression between the PE and MMN intervention arms did not differ (Figure 4).

No differences were observed in the relative gene expression of *DMT1*, *FPN1*, hepcidin, and *ZP* (*P* > 0.55) (**Supplemental Figure 1**A–D) in the placentas between intervention arms.

#### Effect of supplementation on mRNA expression of genes encoding for placental zinc transporter proteins.

**FIGURE 5:**
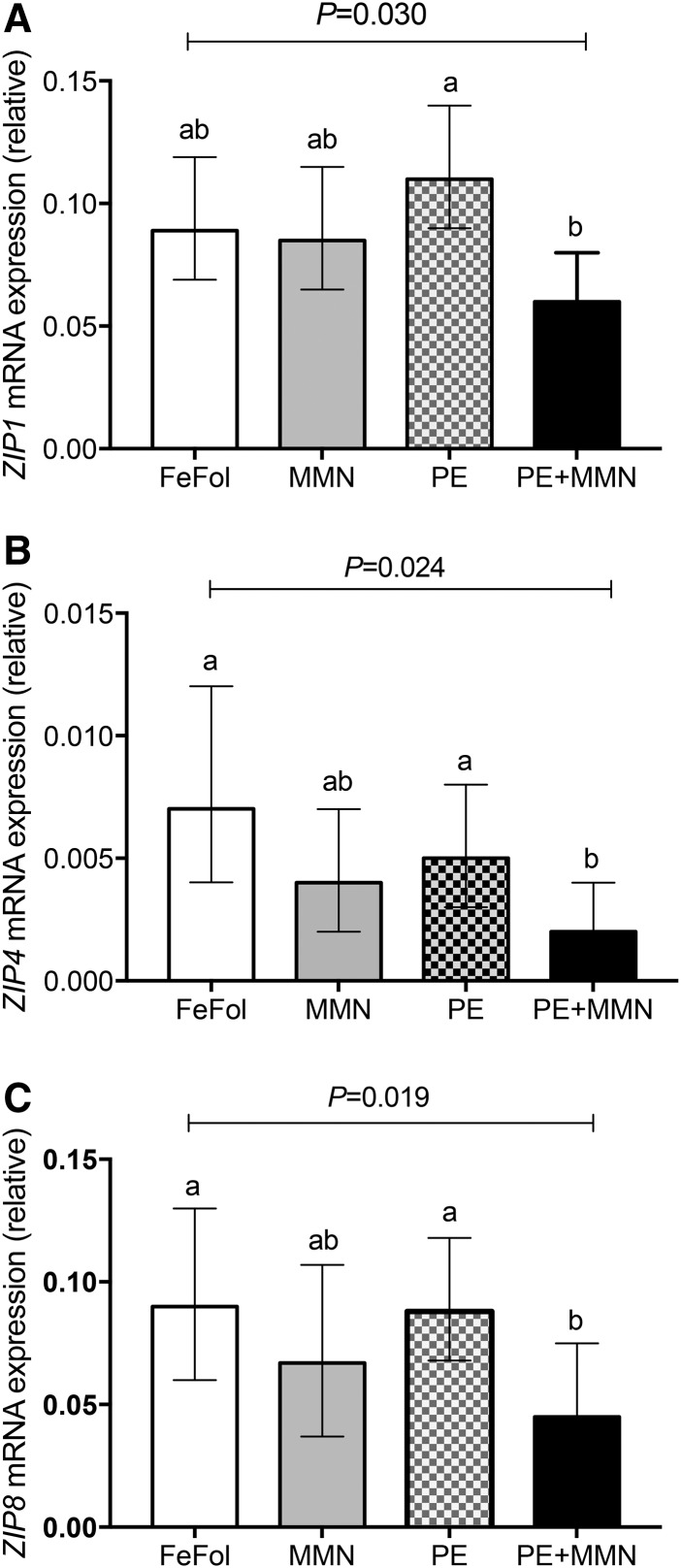
Placental mRNA expression of the zinc uptake genes *ZIP1* (A), *ZIP4* (B), and *ZIP8* (C), relative to the ubiquitin housekeeper, by intervention arm. Placental samples with an RNA integrity number >4 were analyzed. Log-transformed data were analyzed by 1-factor ANOVA, adjusted for gestational age and maternal BMI at booking, season of birth, and maternal parity. Values are back-transformed adjusted means and their corresponding back-transformed 95% CIs (*n* = 69–73). Means without a common letter differ, *P* < 0.05. FeFol, iron and folic acid; MMN, multiple micronutrient; PE, protein energy; *ZIP*, zrt, irt-like protein.

We measured mRNA expression of the intracellular zinc uptake genes, zrt, irt-like protein (ZIP) 1 (*ZIP1*), *ZIP4*, *ZIP8*, and *ZIP14*, and the intracellular zinc exit genes, zinc transporter (ZNT) 1 (*ZNT1*) and *ZNT4*, in the placentas. *ZIP1* mRNA expression was 85% (95% CI: 23%, 177%; *P* = 0.003) lower in the PE+MMN intervention arm than in the PE arm (**Figure 5A**). No differences between the other arms were observed.

The mRNA expression of *ZIP4* was 205% lower (95% CI: 46%, 538%; *P* = 0.003) and 131% (95% CI: 11%, 380%; *P* = 0.025) lower in the PE+MMN arm than in FeFol and PE intervention arms, respectively (Figure 5B). No other differences were observed.

Similarly, the mRNA expression of the zinc importer gene *ZIP8* was 100% (95% CI: 22%, 228%; *P* = 0.006) and 96% (95% CI: 20%, 219%; *P* = 0.007) lower in the PE+MMN arm than in the FeFol and PE intervention arms, respectively, but no other differences were observed (Figure 5C). The mRNA expression of *ZIP14* showed a trend similar to that of *ZIP4* and *ZIP8*, but not to a statistically significant level (*P* = 0.097) (**Supplemental Figure 2**A). However, no statistically significant difference was found in the mRNA expression of the zinc exit genes *ZNT1* and *ZNT4* between the placentas from the intervention arms (Supplemental Figure 2B, C).

#### Effect of supplementation on cord blood iron concentrations.

**FIGURE 6:**
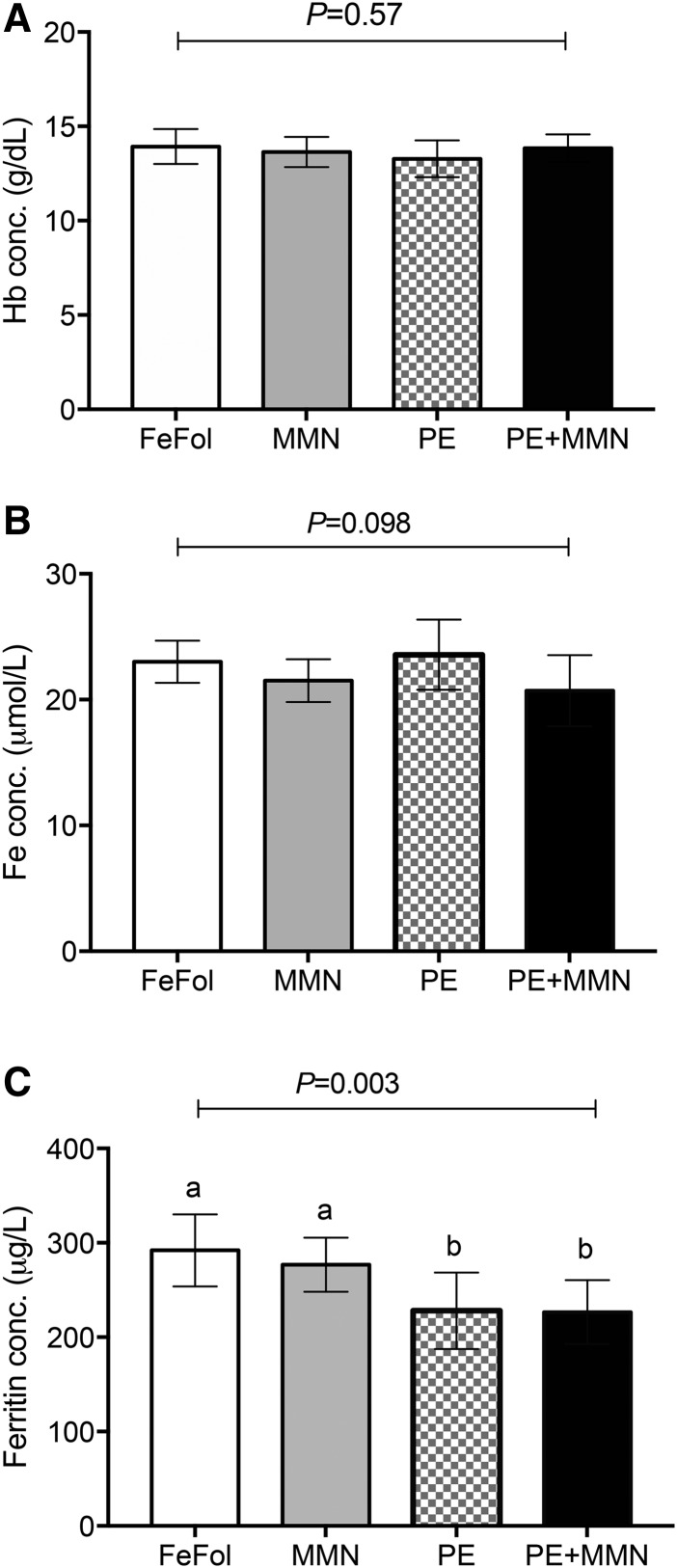
Cord blood concentrations of Hb (A), iron (B), and ferritin (C), by intervention arm. Data were analyzed by 1-factor ANOVA, adjusted for gestational age and maternal BMI at booking, season of birth, and maternal parity. Values are adjusted means ± SEMs (*n* = 50–61). Means without a common letter differ, *P* < 0.05. conc., concentration; FeFol, iron and folic acid; Hb, hemoglobin; MMN, multiple micronutrient; PE, protein energy.

Mean cord blood hemoglobin and iron concentrations were not different between the intervention arms (*P* > 0.098) (**Figure 6A, B**). However, the mean ferritin concentration was relatively lower (*P* < 0.021) in the cord blood of infants born to women randomly assigned to the LNS arms (PE and PE+MMN) than in those born to mothers in the FeFol and MMN intervention arms (Figure 6C).

## Discussion

In this study we examined the effect of nutritional interventions during pregnancy on maternal iron and zinc status and gene expression of placental iron and zinc transporter proteins in a cohort of rural African women. Our data is, to our knowledge, the first evidence from a human population with marginal nutritional status in which alterations to maternal iron and zinc status, as a consequence of nutritional supplementation, results in changes in the gene expression of transporter proteins responsible for iron and zinc uptake across the placenta. Low maternal iron values in late pregnancy, as a consequence of the unexpectedly low iron values of women randomly assigned to the 2 LNS groups, resulted in higher placental gene expression of the primary iron uptake protein *TfR1* when compared with their corresponding non-LNS equivalent (no MMN and with MMN groups). While fetal (cord blood) hemoglobin and iron data suggest that this placental adaptation (increased *TfR1* gene expression) helped improve fetal iron values, fetal ferritin data indicate lower iron stores for the infants. Furthermore, our data suggest that the absence of prenatal supplemental zinc resulted in high gene expression of zinc importer proteins in the placenta, signaling inadequate fetal zinc acquisition to trigger the placental response to increase supplies.

During development, the fetus depends entirely on maternal iron supplied through the placenta for its iron requirement (16); this supply is facilitated by the primary iron uptake protein, TfR1, located on the placental apical membrane. The ferric iron taken up from the maternal circulation by placental TfR1 is transported across the placenta with the help of a series of iron transporter proteins, including DMT1, FPN1, and ZP, before being incorporated onto fetal transferrin. Thus, regulation of the protein expression and activity of these transporters, most importantly TfR1, is a critical aspect of fetal iron accretion. Research in rats (22) and limited data from human observational studies (23–25) has shown increased expression of placental TfR1 in response to maternal iron deficiency. In our study, iron supplementation in LNS format produced an unexpectedly lower maternal iron status in late pregnancy compared with iron supplementation in tablet formulation. The mean plasma concentration of ferritin in the LNS-treated groups was 14.1 μg/L by 30 wk of gestation; 68% of women had a plasma ferritin concentration <15 μg/L, indicating diminished iron stores. In addition, the plasma concentrations of hepcidin and iron were lower during late pregnancy, indicating a greater systemic demand for iron (26). Plasma sTfR, transferrin, and UIBC, however, were higher during late pregnancy in the LNS groups, indicating iron deficiency and increased cellular iron demand.

Our data indicate that, in situations of reduced maternal iron concentrations, the gene expression of placental *TfR1* protein is higher. Upregulation of placental *TfR1* mRNA expression suggests an increased intrauterine iron demand and a placental adaptation to try and increase supplies to the fetus. Although this placental adaptation to maternal iron deficiency helped improve cord hemoglobin and circulating iron concentrations, cord ferritin status (reflecting infant iron stores) was significantly lower in the groups with lower maternal iron values, suggesting that the adaptation might not be completely successful. Inadequate iron accretion in utero might have an implication on the development of fetal organs such as the brain and kidneys. In the rat iron deficiency model, placental TfR1 expression is increased, together with its protein turnover and cycle rate, suggesting an intrauterine stressor, and the affected fetuses had smaller kidneys than normal and higher blood pressure in postnatal life (27–29). Furthermore, low iron stores in early infancy may compromise postnatal development, especially in populations with high rates of exclusive breastfeeding, as in The Gambia, given the known low amounts of iron in breast milk.

Despite consistent concentrations of iron (60 mg/d) in all intervention arms, we observed unexpectedly lower iron amounts at 30 wk of gestation in the 2 groups receiving LNS. A previous trial of LNS against other formulations of micronutrient supplementation in infants had shown LNS to be comparable to iron status (30). Most trials that had used LNS as an intervention used small-quantity lipid-based supplements (typically 20 g/d; ∼108 kcal) (10, 11). In this trial, women receiving LNS were provided with a daily dose of 140 g (∼746 kcal). One possible explanation for this finding is that women in the FeFol and MMN arms benefitted from additional iron gained through normal dietary consumption, which might be negated by the high caloric content of the LNS intervention in this trial. In this rural Gambian setting, however, where dietary intake of meat and other foods containing large amounts of iron is low, we do not consider this explanation likely. We think it is more likely a consequence of a poorer compliance to supplementation in the LNS groups. Compliance data collected as part of the main ENID trial indicate higher compliance among women receiving the tablet formulation than among those receiving the LNS formulation (94% compared with 82%, respectively; *P* < 0.0001; SE Moore, unpublished results, 2017). This pattern was consistent within the current subgroup of women (94% compared with 81%; *P* < 0.0001). If women were failing to consume the complete dose of LNS each day, they would not receive the allocated daily iron supply (60 mg/d).

Whereas the importance of zinc for fetal development has been recognized (3, 31), less is currently known about the mechanism of placental transport of zinc to the fetus. As in duodenal epithelial cells, however, the placental syncytiotrophoblast is assumed to use the 2 zinc transporter families, zinc importer protein family ZIP (*SLC39*) and zinc exporter protein family ZNT (*SLC30*) to deliver zinc (32, 33). At the level of the duodenum, some of these transporters are responsive to dietary zinc intake and show a tonic response to changing zinc levels, increasing deficiency and dropping as concentrations are restored (34–36). Our data suggest that the unavailability of dietary zinc in a prenatal supplement (FeFol and PE) is associated with increased gene expression of zinc uptake proteins in the placenta, presumably to try and meet fetal zinc needs.

The key strength of our study is that the trial design allowed us to directly interrogate the placental response to maternal iron and zinc concentrations in a population with high rates of micronutrient deficiency. The large sample size and samples collected prospectively during pregnancy and at delivery allowed a detailed investigation of the maternal response to supplementation and the impact this had on placental expression of both iron and zinc transporters. Limitations include the lack of early gestation or cord blood measures of zinc values, and a later infancy measure of both iron and zinc values. Further, whereas we indicate gene expression levels, we were not able to quantify protein levels or to indicate the functional capacity of these transport proteins.

In conclusion, our data indicate that alterations in maternal iron and zinc concentrations, as a consequence of nutritional supplementation during pregnancy, have a regulatory effect on the gene expression of intrauterine iron and zinc transporter proteins in the placenta, with consequences for fetal nutritional status. Despite placental upregulation of TfR1 in response to maternal deficiency, however, fetal iron markers indicate that infant status may remain suboptimal in situations of maternal iron deficiency. Further work is warranted to replicate these observations and to explore the long-term impact of this apparent placental regulation on both maternal and infant micronutrient status. Continued attention should focus on interventions to improve micronutrient concentrations in women of reproductive age in settings of nutritional vulnerability.
